# A Comprehensive Analysis of the *Fowleria variegata* (Valenciennes, 1832) Mitochondrial Genome and Its Phylogenetic Implications within the Family Apogonidae

**DOI:** 10.3390/genes14081612

**Published:** 2023-08-11

**Authors:** Jiaqiao Wang, Weiyi He, Hao Huang, Danyun Ou, Lei Wang, Jun Li, Weiwen Li, Site Luo

**Affiliations:** 1 Fisheries College of Ji Mei University, Xiamen 361000, China; 2 Fujian Provincial Key Laboratory of Marine Fishery Resources and Eco-Environment, Xiamen 361000, China; 3 Third Institute of Oceanography, Ministry of Natural Resources, Xiamen 361005, China; 4 School of Life Sciences, Xiamen University, Xiamen 361102, China

**Keywords:** *Fowleria variegata*, mitochondrial genome, phylogeny analysis

## Abstract

Controversies surrounding the phylogenetic relationships within the family Apogonidae have persisted due to the limited molecular data, obscuring the evolution of these diverse tropical marine fishes. This study presents the first complete mitochondrial genome of *Fowleria variegata*, a previously unrepresented genus, using high-throughput Illumina sequencing. Through a comparative mitogenomic analysis, *F. variegate* was shown to exhibit a typical genome architecture and composition, including 13 protein-coding, 22 tRNA and 2 rRNA genes and a control region, consistent with studies of other Apogonidae species. Nearly all protein-coding genes started with ATG, while stop codons TAA/TAG/T were observed, along with evidence of strong functional constraints imposed via purifying selection. Phylogenetic reconstruction based on maximum likelihood and Bayesian approaches provided robust evidence that *F. variegata* forms a basal lineage closely related to P. trimaculatus within Apogonidae, offering novel perspectives into the molecular evolution of this family. By generating new mitogenomic resources and evolutionary insights, this study makes important headway in elucidating the phylogenetic relationships and mitogenomic characteristics of Apogonidae fishes. The findings provide critical groundwork for future investigations into the drivers of diversification, speciation patterns, and adaptive radiation underlying the extensive ecological diversity and biological success of these marine fishes using phylogenomics and population genomics approaches.

## 1. Introduction

**Figure 1:**
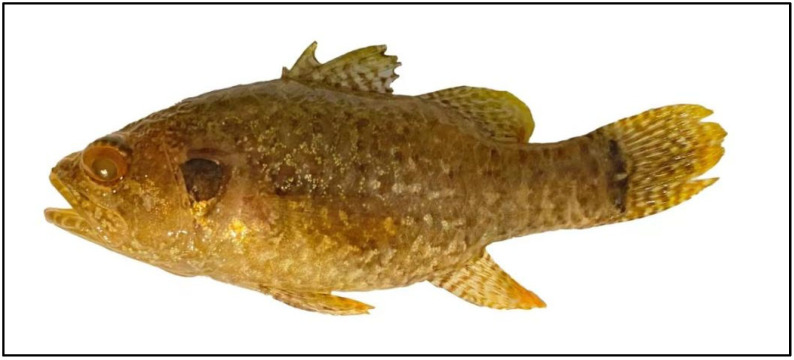
The sample image of *F. variegata*, taken by Weiyi He.

*Fowleria variegata,* commonly called the variegated butterflyfish, is a marine fish species belonging to the family Apogonidae [1,2]. *F. variegata* naturally occurs in tropical waters of the Indo-Pacific region, spanning the Red Sea, eastern African coast, and western Pacific Ocean. *F. variegata* is highly sought-after owing to the vibrant and distinct coloration that makes it a popular choice for aquarium enthusiasts. The *F. variegata* is characterized by its oval-shaped body, which is laterally compressed and highly flattened. It has a small mouth, a long snout, and a continuous dorsal fin that runs along its back [3]. The coloration of this species is its most striking feature, with a pattern of alternating bands of black, white, and yellow on its body. These vibrant colors not only serve as a form of camouflage, but also make *F. variegata* an attractive sight on coral reefs. In terms of habitat, *F. variegata* is primarily found in coral-rich areas, particularly around reef slopes and outer reef zones. It prefers shallow depths of up to 40 m, where it can easily access its main food source—coral polyps [4]. This species has a specialized diet, primarily feeding on small invertebrates and zooplankton, which it extracts from the coral using its small, beak-like mouth (Figure 1). The reproductive behavior of the *F. variegata* involves pair bonding, in which a male and a female form a monogamous partnership. They engage in courtship displays and territorial behavior in order to establish their breeding grounds. The female fish lays a large number of pelagic eggs, which are fertilized by the male. The eggs are then released into the water column and left to fend for themselves. While *F. variegata* is not currently listed as endangered, it does face various threats due to human activities and environmental changes. Habitat destruction, caused by factors such as coastal development, pollution, and coral bleaching, poses a significant risk to the species. Overfishing for the aquarium trade also impacts its population in certain areas [5]. As a result, conservation efforts are necessary to protect the *F. variegata* and ensure the long-term viability of its habitat.

Mitochondria, present in almost all eukaryotic organisms, play vital roles in regulating energy metabolism, apoptosis, aging, and various diseases, establishing them as essential components within cells [6]. Mitochondrial DNA (mtDNA) is a valuable molecular marker for systematic studies. It is widely used due to its simple structure, rapid evolutionary rate, abundant copies, and ease of isolation. These characteristics make mtDNA a convenient and effective tool for investigating genetic relationships and phylogenetic patterns [7]. Mitochondrial genomes (mitogei nomes) are pivotal in molecular biology research as they provide crucial insights into evolutionary relationships, population history, and genetic diversity [8]. They are extensively employed in species identification, classification, and phylogenetic analysis, enabling the revelation of species’ phylogenetic relationships and aiding in the reconstruction of a genus’s evolutionary tree [9]. Moreover, mitochondrial genomes facilitate the study of gene flow, migration patterns, and genetic diversity among species [10]. However, the absence of mitochondrial genome sequences in species belonging to the genus *Fowleria* is creating a significant gap in molecular biology research. Scientists are unable to utilize mitochondrial genomes for species identification, classification, and evolutionary analysis, leading to an incomplete understanding of the phylogenetic relationships and population history within the genus. This deficiency may give rise to misconceptions regarding species relationships and confusion in the taxonomic positioning of the entire genus.

This study presents a comprehensive analysis of the mitochondrial genome (mitogenome) of *F. variegata*, a species belonging to the genus *Fowleria*. We have successfully assembled the complete mitogenome of *F. variegata* using paired-end (PE 150) sequencing technology. This achievement not only enhances our understanding of *F. variegata*’s genetic composition, but also provides valuable insights into the phylogenetic relationships within the broader family Apogonidae. The mitogenomic data presented in this study represent a significant expansion of the existing knowledge of the family Apogonidae. This study presents the first complete mitogenome for any species in the genus *Fowleria*, providing a robust dataset that can be used to investigate the phylogeny of the family Apogonidae in greater detail. The availability of complete mitogenomes of the Apogonidae species strengthens our ability to explore the evolutionary history and genetic diversity of this taxonomic group. The findings presented in this research article will lay the foundation for further studies on the genus *Fowleria* species and contribute significantly to the broader field of fish phylogenetics. This work represents a major advance in our understanding of the evolutionary history of Apogonidae and will help to shed light on the relationships between this diverse group of fishes.

## 2. Materials and Methods

### 2.1. Ethical Approval for Research Protocols

Animal handling and experimentation protocols adhered to the guidelines and regulations for laboratory animal care in China. The research protocols were approved by the institutional animal care and use committee in accordance with the ethical regulations for animal studies issued by the China Council on Animal Care.

### 2.2. Experimental Fish and Sampling

Genomic DNA was extracted from the collected *F. variegata* sample using the TIANamp Genomic DNA Kit (TIANGEN, Beijing, China), following the manufacturer’s protocol. Approximately 0.2 μg of the extracted DNA was fragmented into ~350 bp pieces to generate overlapping short fragments suitable for sequencing. The sequencing library was constructed in accordance with to the kit guidelines. It involved fragment end-repair, adapter ligation, and PCR enrichment. The prepared library was sequenced on an Illumina Nova 6000 platform, generating 6 Gb short reads with substantial coverage of the F. variegata genome. The TIANamp kit is a reliable extraction method widely used in molecular biology research. Following the standardized protocols ensured high-quality DNA extraction and library construction for optimal sequencing results.

### 2.3. F. variegata Mitogenome Assembly and Annotation

The *F. variegata* mitogenome was assembled using the GetOrganelle pipeline with default parameters, an approach that ensures accurate mitogenome assembly [11]. The pipeline extracted seed reads from the ‘animal_mt’ database to initiate assembly. After assembly completion, the short reads were aligned back to the mitogenome using BWA in order to evaluate coverage and validate the accuracy of the assembly [12]. Pilon was then utilized to further refine the assembly [13]. This step enhanced the accuracy and overall quality of the mitogenome assembly. The integrated use of BWA alignment and Pilon polishing played a key role in improving the assembly and reducing potential errors or inconsistencies.

Following the assembly process, the *F. variegata* mitogenome was annotated to identify various genetic elements. The identification of protein-coding genes (PCGs) was carried out by comparing them to a reference mitogenome using Mitoz v3.4 [14]. This comparison helped to determine the presence and arrangement of these important genetic components. The annotated *F. variegata* mitogenome was generated using MITOS, a widely utilized tool for mitogenome annotation (http://mitos.bioinf.uni-leipzig.de/index.py, accessed on 3 September 2022) [14]. MITOS accurately identified the transfer RNAs (tRNAs) and ribosomal RNAs (rRNAs) encoded in the mitogenome. Precise annotation of these functional RNAs is important as they have key roles in protein synthesis and mitochondrial function.

Circular maps of the *F. variegata* mitogenome were generated using OGDraw in order to visualize the organization and arrangement of genomic features (https://chlorobox.mpimp-golm.mpg.de/OGDraw.html, accessed on 3 September 2022) [15]. The maps illustrate the positions of all genes, tRNAs, and rRNAs in the mitogenome, providing a comprehensive overview of its structure.

### 2.4. Assessment of Sequence Properties

The nucleotide composition, codon usage, and relative synonymous codon usage (RSCU) of the *F. variegata* mitogenome were analyzed using CodonW [16]. This shed light on the nucleotide makeup and codon preferences of the mitogenome. Nucleotide diversity (Pi) and Ka/Ks ratios for the 13 mitochondrial protein-coding genes (PCGs) in Apogonidae were calculated using DnaSP in order to assess genetic variation patterns [17]. Sliding window analyses of the PCGs were also conducted in DnaSP using 100 bp windows with 25 bp steps in order to examine diversity within PCGs. Additionally, genetic distances were estimated using the Kimura-2 parameter (K2P) model in MEGA in order to determine evolutionary relationships. Combining codon usage analysis, Pi, Ka/Ks ratios, and K2P distances enabled us to obtain comprehensive insights into the mitogenomic diversity and evolution of Apogonidae.

### 2.5. Phylogenetic Analyses

To determine phylogenetic relationships within Apogonidae, the 13 concatenated mitochondrial PCGs from *F. variegata* and other Apogonidae species (Table 1) were aligned using MAFFT [18]. ModelFinder identified the optimal evolutionary model (GTR + F + R6) based on the Akaike Information Criterion [19]. This model balanced accuracy and complexity. Maximum likelihood analysis was conducted using IQ-TREE with 1000 ultrafast bootstraps [20]. Bayesian inference employed MrBayes with two independent MCMC runs of 50 million generations, sampling every 1000 generations until convergence [21]. The first 10% of trees were discarded as burn-in before computing a consensus tree. The bootstraps and posterior probabilities provided statistical support to the evaluation of topology robustness. Combining maximum likelihood and Bayesian approaches enabled a robust phylogenomic assessment of the evolutionary relationships in Apogonidae to be performed.

## 3. Observations and Insights

### 3.1. Genomic Organization and Nucleotide Composition

**Figure 2:**
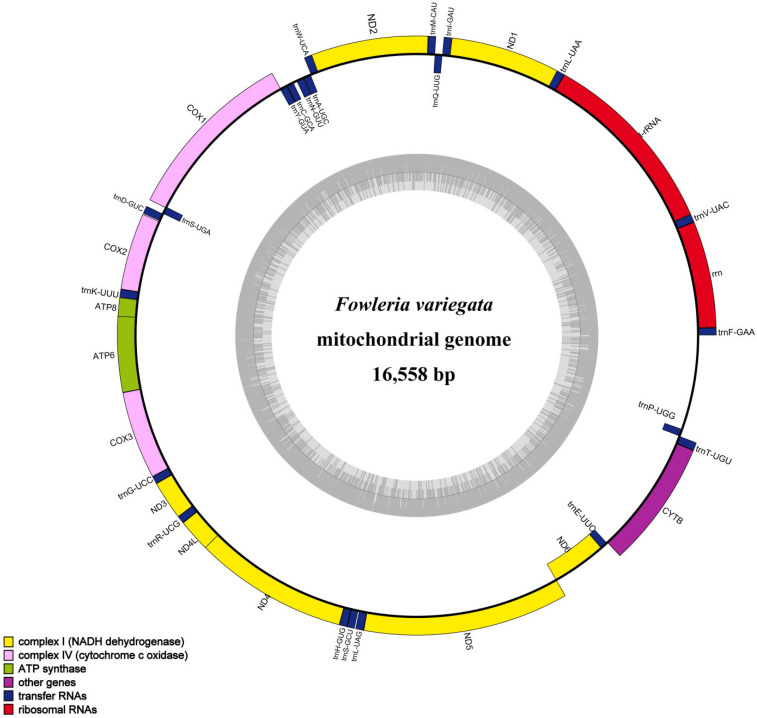
A circular map of the *F. variegata* mitochondrial genome is shown, with the outer circle denoting the heavy (H) strand and the inner circle denoting the light (L) strand. The inner gray circle illustrates the GC and AT content distribution, where darker regions indicate higher GC content and lighter regions indicate higher AT content.

**Figure 3:**
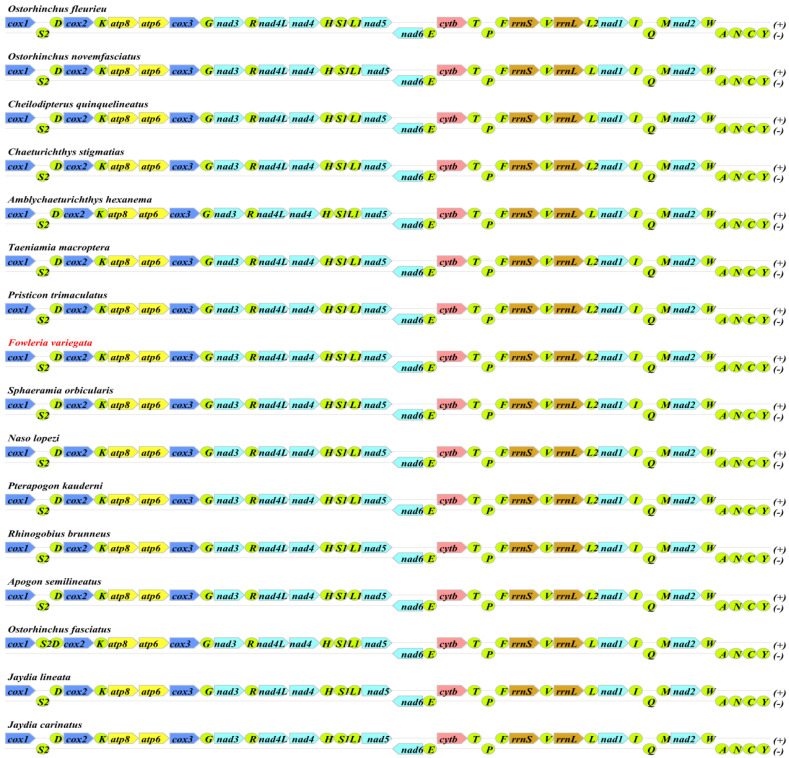
Mitochondrial Genomes of Apogonidae Species Analyzed. Note: *F. variegata* highlighted in red.

The *F. variegata* mitogenome was characterized as a 16,558-base-pair circular molecule. The analysis of its nucleotide composition revealed 28.14% A, 25.64% C, 18.81% G, and 28.41% T, reflecting an AT bias (56.55%) consistent with that of other Apogonidae species. The mitogenome contains 13 protein-coding genes, 22 transfer RNAs, 2 ribosomal RNAs, and a control region with high AT content (Figure 2, Table 2). The shortest tRNAs were tRNAPhe, tRNACys, and tRNASer at 69 bp, while the longest were tRNALeu, tRNAAsn, and tRNALeu at 74 bp. The 896 bp control region lies between tRNAPro and tRNAPhe. Our comparative analysis showed remarkable similarity with result from other Apogonidae, with variations of 10–221 bp primarily seen in control region-associated genes (Figure 3, Table 1). Such variations indicate potential divergence and evolution patterns in Apogonidae.

In summary, characterization of the *F. variegata* mitogenome revealed typical features including AT bias and conserved RNAs and genes, highlighting their functional significance. Variations among Apogonidae species point to a complex interplay between conservation and adaptation. Further investigation of these variations will provide deeper insights into mitogenomic diversity and evolution in Apogonidae.

### 3.2. Analysis of Mitochondrial Protein-Coding Genes

The F. variegata mitogenome contains a typical set of 28 genes—9 encoded on the L-strand (nad6, trnQ, trnA, trnN, trnC, trnY, trnS, trnE, and trnP) and 19 on the H-strand (atp6, atp8, cox1-3, cob, nad1-5, l-rRNA, s-rRNA, trnD-G, trnH-M, trnR-V, and trnW). The gene order closely matched that of previous Apogonidae mitogenomes (Figure 3) [22,23,25,26,27,28,29,30,31,32]. The conservation of gene composition and arrangement indicates a shared evolutionary history and suggests that these genes can contribute to phylogenetic resolution at the family level of Apogonidae species. The observed similarities in gene order and orientation between this study and previous investigations imply the potential utility of these genes in future phylogenetic studies within the family Apogonidae.

**Figure 4:**
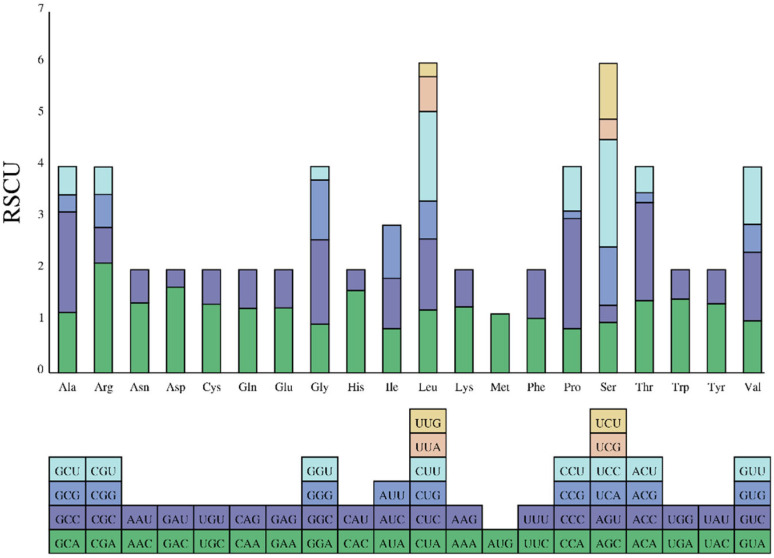
Codon usage in *F. variegata* mitochondrial protein-coding genes.

The *F. variegata* mitogenome contains 3824 codons across 13 protein-coding genes (Figure 3). Codon usage patterns provide insights into gene expression, mRNA stability, and phylogenetic relationships [33]. Leucine, alanine, threonine, phenylalanine, isoleucine, and proline were the most prevalent amino acids in the codons, representing 5.05%, 4.65%, 3.48%, 3.37%, 3.37%, and 3.19%, respectively (Figure 4, Table S1). In contrast, cysteine and proline were the rarest at shares of 0.24%. The biased codon usage underscores their utility in elucidating evolution and phylogeny in *F. variegata*.

**Figure 5:**
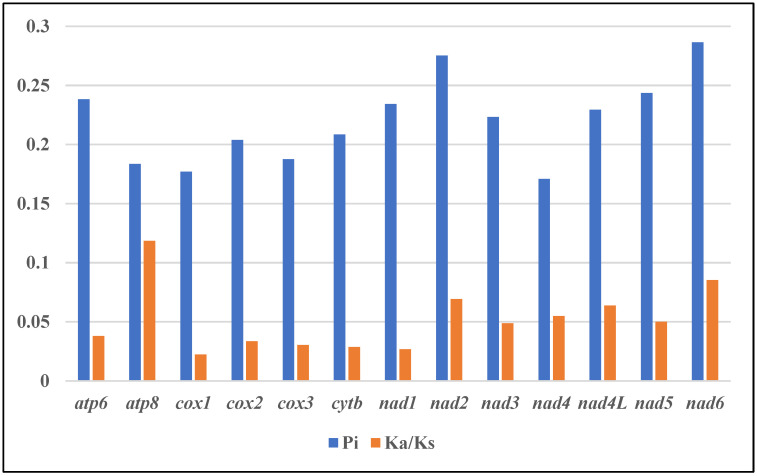
Genetic diversity and evolutionary dynamics of mitogenomes in this study.

All 13 mitogenome protein-coding genes exhibited Ka/Ks ratios below 1 (0.02–0.12), which indicated purifying selection (Figure 5, Table S2) [34]. The *atp8*, *nad6*, *nad2* and *nad4L* genes showed relatively lower Ka/Ks, suggesting weaker evolutionary pressures and the retention of more non-synonymous mutations. Cox1 displayed the lowest Ka/Ks ratio, reflecting stronger selection and functional constraints [35]. This is significant since mitochondrial DNA encodes essential respiratory components and governs inheritance, making the mitogenome susceptible to the accumulation of deleterious mutations [36]. The use of strong purifying selection on cox1 eliminates such mutations, rendering it ideal for application to Apogonidae phylogeny. Consequently, these genes likely contribute to phylogenetic resolution at the genus level within Apogonidae, providing insights into evolutionary relationships and divergence [37].

Nucleotide diversity (π) quantifies the average differences between two randomly selected sequences in a gene or genomic region. It represents a fundamental genetic parameter that measures the extent of genetic variation or diversity within a population. Higher π values denote greater diversity in the nucleotide sequences of a specific region. Assessing nucleotide diversity allows researchers to evaluate the level of genetic variation present.

Nucleotide sequence alignments of 13 PCGs from 12 Apogonidae mitogenomes were analyzed in order to identify DNA polymorphisms (Figure 4, Table S2). This revealed the nucleotide diversity (π) between the genes in these mitogenomes. Interestingly, the nad6 gene exhibited the highest nucleotide diversity, with a π value of 0.286. It was followed closely by *nad2* (0.275), *nad5* (0.243), and *nad6* (0.238). On the other hand, the *nad4* (0.170) and *cox1* (0.177) genes displayed the lowest nucleotide diversity values within the dataset. To further characterize the genetic distances between the sequences, we analyzed the mean genetic distances for these genes (Table S3). Mirroring the nucleotide diversity, *nad6*, *nad2*, *nad4* and *nad5* showed higher genetic distances of 0.29, 0.28, 0.28 and 0.25 respectively, implying greater sequence divergence. In comparison, *cox1*, *cox3* and *atp8* exhibited lower distances of 0.18, 0.19 and 0.19 respectively, denoting relatively lower divergence.

These findings offer insights into genetic diversity and sequence divergence in protein-coding genes among Apogonidae mitogenomes. The identification of genes with high nucleotide diversity and genetic distances, such as *nad6*, *nad2*, *nad4*, and *nad5*, suggests that these genes may be subjected to selective pressures or evolutionary forces that contribute to their higher variability. Further exploring the functional roles of these genes and their evolutionary implications in Apogonidae would improve our understanding of genetic diversity and adaptation in this family.

### 3.3. Phylogenetic Analyses

To ensure robust phylogenetic analysis, our dataset was expanded to 16 mitogenomes. This included 12 from Apogonidae as the focal family and 3 from Gobiidae plus 1 from Acanthuridae as outgroups. These reference mitogenomes were retrieved from the NCBI RefSeq database, with data updated as of 17 June 2023.

**Figure 6:**
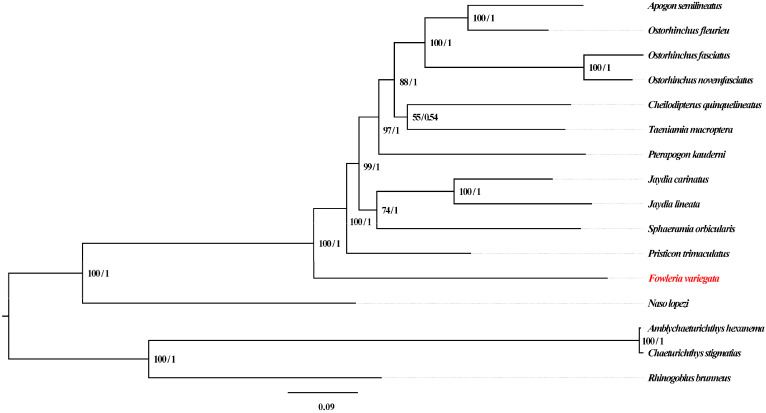
Phylogenetic Reconstruction of 16 Fish Mitogenomes Using 13 PCGs. Note: *F. variegata* highlighted in red.

Phylogenetic relationships were investigated using both maximum likelihood (ML) and Bayesian inference (BI) analyses (Figure 6). *F. variegata* occupied a basal position within Apogonidae and showed affinity to *P. trimaculatus*, in accordance with a prior study performed using three mitochondrial genes (*nad1*, *nad2* and *cox1*) [5]. However, previous mitogenomic analyses lacked genus *Fowleria* representation [22,23,25,27,32]. Our study provides the first complete *Fowleria* mitogenome and phylogenetic analysis, addressing this gap. *F. variegata*’s basal status provides insights into Apogonidae evolution, highlighting the need for further analyses with complementary datasets.

The outgroup mitogenomes enabled comprehensive phylogenetic evaluation to be carried out within Apogonidae. However, performing augmentation with data from other genomic regions or analytical approaches would reinforce these findings. Future efforts should focus on validating and expanding these observations in order to advance the understanding of evolutionary dynamics and relationships in Apogonidae.

## 4. Summary

This study presents the first complete mitochondrial genome of *F. variegata* using short-read sequencing technology, making a valuable addition to the limited genomic resources for the study of this genus. Through comparative genomic analysis, *F. variegata* was found to possess the typical mitogenomic composition of 13 protein-coding, 22 tRNA, and 2 rRNA genes along with a control region, in accordance with other species in the family Apogonidae. The performance of phylogenetic reconstruction using maximum likelihood and Bayesian inference analysis provided robust support for the basal position of *F. variegata*, closely related to *P. trimaculatus*, within family Apogonidae. These findings significantly enhance current understanding of the molecular evolution and phylogeny of this commercially and ecologically important perciformes family.

Further analysis of selection pressures and Ka/Ks ratios in protein-coding genes offered new insights into the evolutionary dynamics of Apogonidae mitogenomes. The genes were found to have undergone varying levels of purifying selection, rendering them promising markers for use in future population genetics studies on genetic differentiation, gene flow, and local adaptation. Targeted investigation of the genes under differential evolutionary constraints will help to elucidate population structure, demographic histories, and the impacts of environmental factors on genetic variations.

Moreover, the elucidation of phylogenetic relationships and comparative mitogenomic analysis in this study establishes critical groundwork for future research into the genetic diversity, adaptation and evolutionary trajectories of *F. variegata* and its related species. The integration of expanded molecular datasets, diverse analytical approaches and a solid systematic framework will provide powerful tools for uncovering the intricacies that underly diversification and adaptation in Apogonidae fishes. Findings from such endeavors will offer valuable insights into the drivers of the speciation and biodiversity critical for conserving and managing these tropical marine fishes.

In summary, by generating novel mitogenomic resources and evolutionary perspectives, this study makes important headway in advancing research into the ecological genomics and molecular systematics of an understudied Perciformes group.

## Figures and Tables

**Table 1:** Mitochondrial genome sequences of Apogonidae species from NCBI used in this study.

Genus	Species	Size (bp)	Accession No.	Resource
Apogonidae	*Apogon semilineatus*	16,508 bp	AP005996	[22]
	*Cheilodipterus quinquelineatus*	16,537 bp	NC_040863	[23]
	*F. variegata*	16,558 bp	ON057361	this study
	*Jaydia carinatus*	16,519 bp	MN011548	[24]
	*Jaydia lineata*	16,510 bp	MT363638	[25]
	*Ostorhinchus fasciatus*	16,568 bp	MN728946	[26]
	*Ostorhinchus fleurieu*	16,521 bp	NC_056170	[27]
	*Ostorhinchus novemfasciatus*	16,779 bp	NC_053536	[28]
	*Pristicon trimaculatus*	16,512 bp	AP018928	[29]
	*Pterapogon kauderni*	16,530 bp	AP005997	
	*Sphaeramia orbicularis*	16,458 bp	AP018927	[30]
	*Taeniamia macroptera*	16,513 bp	MN066612	
Acanthuridae	*Naso lopezi*	16,542 bp	AP009163	
Gobiidae	*Amblychaeturichthys hexanema*	18,562 bp	KT781104	
	*Chaeturichthys stigmatias*	18,562 bp	KC495071	[31]
	*Rhinogobius brunneus*	16,500 bp	KT601096	[32]

**Table 2:** Features annotated in the *F. variegata* mitochondrial genome.

Locus	Start	Stop	Size (bp)	Start Coding	Stop Coding	Strand
*tRNA*Phe	1	69	69			H
*12S rRNA*	69	1024	956			H
*tRNA*Val	1024	1096	73			H
*16S rRNA*	1097	2784	1688			H
*tRNA*Leu	2784	2857	74			H
*nad1*	2816	3829	1014	ATG	TAA	H
*tRNA*Ile	3831	3901	71			H
*tRNA*Gln	3900	3971	72			L
*tRNA*Met	3970	4039	70			H
*nad2*	4039	5082	1045	ATG	T	H
*tRNA*Trp	5084	5156	73			H
*tRNA*Ala	5160	5229	70			L
*tRNA*Asn	5230	5303	74			L
*tRNA*Cys	5338	5406	69			L
*tRNA*Tyr	5406	5476	71			L
*cox1*	5468	7027	1560	GTG	TAA	H
*tRNA*Se^r^	7030	7101	72			L
*tRNA*Asp	7103	7175	73			H
*cox2*	7181	7872	692	ATG	T	H
*tRNA*Lys	7872	7944	73			H
*atp8*	7945	8113	169	ATG	TAA	H
*atp6*	8103	8787	685	ATG	TAA	H
*cox3*	8786	9572	787	ATG	T	H
*tRNA*Gly	9571	9641	71			H
*nad3*	9641	9992	352	ATG	TAG	H
*tRNA*Arg	9990	10,059	70			H
*nad4l*	10,059	10,356	298	ATG	TAA	H
*nad4*	10,349	11,730	1382	ATG	TAG	H
*tRNA*His	11,730	11,799	70			H
*tRNA*Ser	11,799	11,867	69			H
*tRNA*Leu	11,875	11,948	74			H
*nad5*	11,948	13,787	1840	ATG	TAG	H
*nad6*	13,783	14,305	523	ATG	TAA	L
*tRNA*Glu	14,306	14,375	70			L
*cob*	14,380	15,521	1142	ATG	T	H
*tRNA*Thr	15,521	15,593	73			H
*tRNA*Pro	15,593	15,663	71			L

## Data Availability

The genome sequence data that support the findings of this study are openly available in GenBank of NCBI at (https://www.ncbi.nlm.nih.gov/, accessed on 5 April 2023) under accession no ON057361. The associated BioProject, SRA, and Bio-Sample numbers are PRJNA924343, SAMN32758635, SRR23095444, respectively.

## Supplementary Materials

The following supporting information can be downloaded at: https://www.mdpi.com/article/10.3390/genes14081612/s1, Supplementary Table S1: Amino acid composition and relative synonymous codon usage in the *F. variegata* mitogenome; Supplementary Table S2: Nucleotide diversity, Ka, and Ks values for protein-coding genes; Supplementary Table S3: Genetic distances based on 13 protein-coding genes.
